# Multimorbidity clusters in patients with chronic obstructive airway diseases in the EpiChron Cohort

**DOI:** 10.1038/s41598-021-83964-w

**Published:** 2021-02-26

**Authors:** Jonás Carmona-Pírez, Beatriz Poblador-Plou, Ignatios Ioakeim-Skoufa, Francisca González-Rubio, Luis Andrés Gimeno-Feliú, Jesús Díez-Manglano, Clara Laguna-Berna, Jose M. Marin, Antonio Gimeno-Miguel, Alexandra Prados-Torres

**Affiliations:** 1 EpiChron Research Group, Aragon Health Sciences Institute (IACS), IIS Aragón, Miguel Servet University Hospital, 50009 Zaragoza, Spain; 2 Health Services Research on Chronic Patients Network (REDISSEC), ISCIII, 28222 Madrid, Spain; 3 Delicias-Sur Primary Care Health Centre, Aragon Health Service (SALUD), 50009 Zaragoza, Spain; 4 San Pablo Primary Care Health Centre, Aragon Health Service (SALUD), University of Zaragoza, 50003 Zaragoza, Spain; 5 Department of Medicine, Psychiatry and Dermatology, University of Zaragoza, Zaragoza, Spain; 6 Internal Medicine Department, Royo Villanova Hospital, Zaragoza, Spain; 7 Respiratory Service, Miguel Servet University Hospital, Zaragoza, Spain

**Keywords:** Health care, Risk factors, Diseases, Respiratory tract diseases, Asthma, Chronic obstructive pulmonary disease, Medical research, Epidemiology, Statistics

## Abstract

Chronic obstructive airway diseases such as chronic obstructive pulmonary disease (COPD), asthma, rhinitis, and obstructive sleep apnea (OSA) are amongst the most common treatable and preventable chronic conditions with high morbidity burden and mortality risk. We aimed to explore the existence of multimorbidity clusters in patients with such diseases and to estimate their prevalence and impact on mortality. We conducted an observational retrospective study in the EpiChron Cohort (Aragon, Spain), selecting all patients with a diagnosis of allergic rhinitis, asthma, COPD, and/or OSA. The study population was stratified by age (i.e., 15–44, 45–64, and ≥ 65 years) and gender. We performed cluster analysis, including all chronic conditions recorded in primary care electronic health records and hospital discharge reports. More than 75% of the patients had multimorbidity (co-existence of two or more chronic conditions). We identified associations of dermatologic diseases with musculoskeletal disorders and anxiety, cardiometabolic diseases with mental health problems, and substance use disorders with neurologic diseases and neoplasms, amongst others. The number and complexity of the multimorbidity clusters increased with age in both genders. The cluster with the highest likelihood of mortality was identified in men aged 45 to 64 years and included associations between substance use disorder, neurologic conditions, and cancer. Large-scale epidemiological studies like ours could be useful when planning healthcare interventions targeting patients with chronic obstructive airway diseases and multimorbidity.

## Introduction

The World Health Organization (WHO) identifies cardiovascular and respiratory diseases, cancer, and diabetes as the four major groups of non-communicable diseases with the highest prevalence and associated mortality risk^1^. Chronic respiratory conditions contribute significantly to health loss and are responsible for more than 5% of years lived with disability in patients older than 50 years^2^. Chronic obstructive airway diseases affect hundreds of millions of people worldwide. Among them, 400 million people suffer from allergic rhinitis, 300 million from asthma, 210 million have a chronic obstructive pulmonary disease (COPD), and more than 100 million are affected by obstructive sleep apnea (OSA)^3^. Rhinitis and asthma stand out in the early stages of life, with asthma representing the most common chronic disease in children^1^. On the contrary, it seems that COPD and OSA appear later in life and usually in combination with frailty and disability.

Initiatives like Integrated Care Pathways for Airway Diseases (AIRWAYS–ICPs) have developed multi-sectorial care pathways for chronic respiratory diseases in Europe^4^. The complex relationships among chronic obstructive airway diseases have been studied in different ways, from an etiopathogenic and clinical perspective. Although COPD, asthma, rhinitis, and OSA have a different etiology, all these entities share two common pathophysiological characteristics that may partly determine their similar multimorbidity. On the one hand, these are diseases that cause airflow limitation in the lower (COPD and asthma) and upper (rhinitis and OSA) respiratory tract; on the other hand, there is a chronic inflammation of the airways^5–8^.

Another relevant issue is that most of the patients affected by chronic obstructive airway diseases present multimorbidity (i.e., more than one chronic disease)^9^. Multimorbidity is associated with a reduction of the potential benefits of respiratory rehabilitation, lower quality of life, and increased mortality^10–12^. Few studies have addressed multimorbidity in patients with chronic respiratory diseases from an epidemiological and comprehensive approach^13^. As far as we know, there are no studies aimed at identifying different profiles of patients based on their comorbidity and including the combination of the most prevalent chronic obstructive airway diseases. In this context, large-scale population-based studies using clinical information from electronic health records could represent an excellent opportunity to generate real-world evidence on the epidemiology of the multimorbidity of chronic obstructive airway diseases. Investigating the nature of the associations among comorbidities may allow us to create new hypotheses on common underlying pathophysiological mechanisms and to identify groups of patients potentially susceptible to different clinical management, facilitating the development of specific preventive and therapeutic approaches^9^.

This study aimed to explore the existence of multimorbidity clusters in patients with COPD, OSA, asthma, and/or rhinitis using cluster analysis, to clinically describe the clusters obtained, and to analyze their impact on mortality.

## Results

The study population was composed of 127,530 individuals 15 years of age and older with a diagnosis of at least one of the chronic obstructive airway diseases studied, representing 10.2% of the reference population in the EpiChron Cohort. The most frequent chronic obstructive airway disease in both sexes was allergic rhinitis, followed by asthma, COPD, and OSA (Table 1). Allergic rhinitis and asthma were more frequent in women than in men, while COPD and OSA were more common in men. Three in every four patients had at least one co-existing chronic condition, with an average of 3.7 diseases per patient. Women showed a higher morbidity burden compared with men.

**Table 1:** Demographical and clinical characteristics of patients with chronic obstructive airway diseases.

Characteristics	Total (N = 127,530)	Men (N = 61,176)	Women (N = 66,354)	*p* value
**Age interval (N, %)**				< 0.001
15–44 years	54,920 (43.0)	25,952 (42.4)	28,968 (43.7)	
45–64 years	33,743 (26.5)	14,552 (23.8)	19,191 (28.9)	
> 65 years	38,867 (30.5)	20,672 (33.8)	18,195 (27.4)	
Rural residence^a^ (N, %)	44,831 (35.2)	22,548 (36.9)	22,283 (33.6)	< 0.001
Multimorbidity^b^ (N, %)	97,759 (76.6)	44,842 (73.3)	52,917 (79.8)	< 0.001
Mean number of chronic diseases (s.d.)	3.7 (2.8)	3.6 (2.9)	3.8 (2.8)	< 0.001
**Chronic obstructive airway diseases (N, %)**				
Allergic rhinitis	65,043 (51.0)	28,677 (46.9)	36,366 (54.8)	< 0.001
Asthma	43,098 (33.8)	16,411 (26.8)	26,687 (40.2)	< 0.001
COPD	28,214 (22.1)	19,725 (32.2)	8,489 (12.8)	< 0.001
Obstructive sleep apnea	1529 (1.2)	1145 (1.9)	384 (0.6)	< 0.001

*s.d.* standard deviation, *COPD* chronic obstructive pulmonary disease.

^a^Versus urban residence.

^b^Defined as the simultaneous presence of two or more chronic diseases from a list of 114 conditions, including each chronic obstructive airway disease separately.

**Figure 1:**
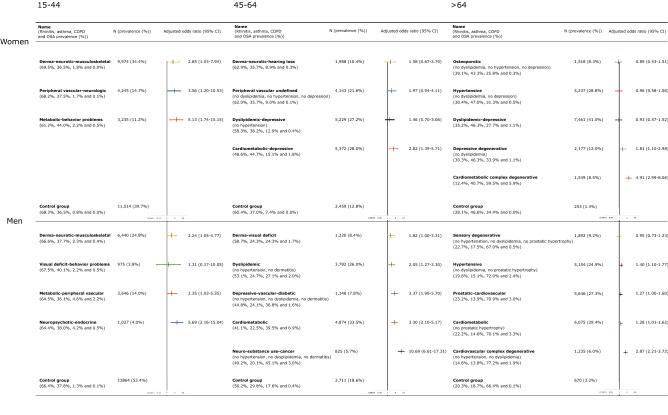
Prevalence of multimorbidity clusters in the study population based on gender and age, and odds ratios of association with mortality. *COPD* chronic obstructive pulmonary disease, *OSA* obstructive sleep apnea.

**Figure 2:**
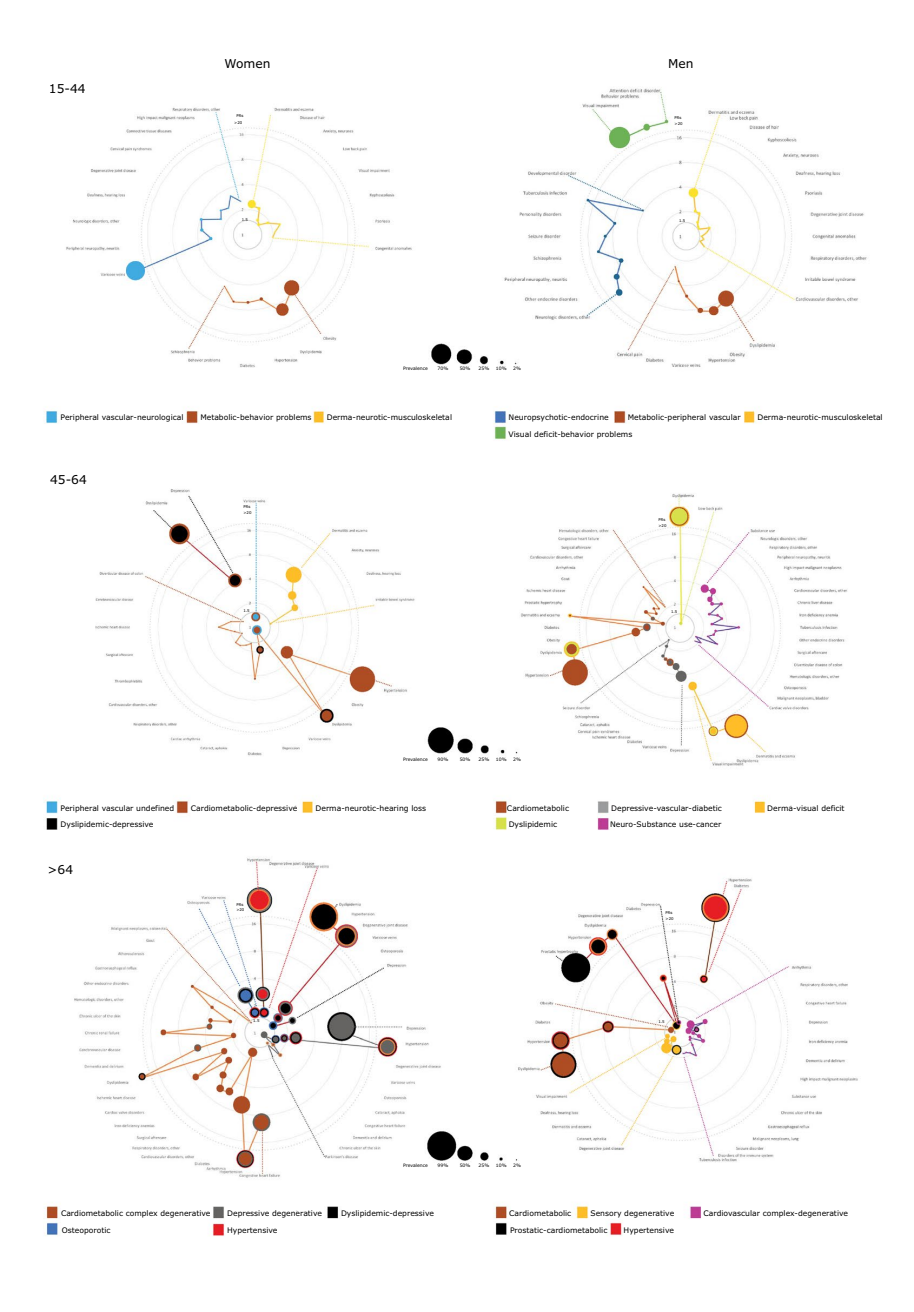
Disease composition of the multimorbidity clusters identified in the study population based on sex and age, according to prevalence ((P); proportional to node size) and prevalence ratios (PRs, we used the lowest PR for each disease). Each cluster has a color, if a node (disease) has more than one color, that disease is included in other clusters.

We included a total of 96,059 patients with multimorbidity in the cluster analysis. Our analysis revealed the existence of systematic associations between chronic diseases. We identified up to twenty-three different multimorbidity clusters in the patients analyzed (Figs. 1 and 2), 11 in women, and 13 in men. The most common chronic comorbidities were cardiometabolic diseases (i.e., dyslipidemia, hypertension, diabetes, and obesity), mental health disorders (i.e., depression, anxiety, and substance use), dermatological diseases (i.e., dermatitis and eczema, and psoriasis), cardiovascular diseases (i.e., ischaemic heart disease, cardiac arrhythmia, and heart failure), and diseases of the musculoskeletal system (i.e., osteoporosis, arthropathy, low back pain, and kyphoscoliosis). The complete output of the cluster analysis is available as supplementary material.

### Multimorbidity clusters in women

We found that both the number and complexity of the multimorbidity clusters increased with age.

In women aged 15–44, we identified three clusters. The derma-neurotic-musculoskeletal one was the most prevalent and included conditions like dermatitis and eczema, disease of hair, anxiety, and low back pain. The metabolic-behavior problems cluster included metabolic diseases (i.e., obesity, dyslipidemia, and diabetes), hypertension, behavior problems, and schizophrenia, and had the highest mortality risk. We also found a peripheral vascular-neurological cluster that included varicose veins as the most prevalent disease, followed by peripheral neuropathy and other neurological disorders.

In women aged 45–64, four clusters were observed. The cardiometabolic-depressive cluster with conditions such as hypertension, obesity, dyslipidemia, varicose veins, and depression was the most prevalent and with the highest mortality risk associated. We also found a dyslipidemic-depressive cluster that included only dyslipidemia and depression, a derma-neurotic-hearing loss cluster with conditions such as dermatitis and eczema, anxiety, deafness, and irritable bowel syndrome, and an undefined peripheral vascular cluster, all of which were not associated with increased mortality risk.

In women aged 65 and older, we described five clusters. The dyslipidemic-depressive cluster with conditions like dyslipidemia, hypertension, degenerative joint disease, osteoporosis, and depression was the most prevalent. The hypertensive group included diseases like hypertension, degenerative joint disease, and varicose veins and was the second most prevalent cluster. The osteoporotic cluster included only osteoporosis and varicose veins and was the less prevalent group. The depressive-degenerative cluster that included depression, hypertension, degenerative joint disease, cataract, dementia, or chronic ulcer of the skin had an increased mortality risk associated. Finally, the cardiometabolic complex-degenerative cluster presented the highest mortality risk, and it included 21 chronic conditions; the most common ones were congestive heart failure, hypertension, arrhythmia, and diabetes.

### Multimorbidity clusters in men

In men aged 15–44, we identified four clusters. The derma-neurotic-musculoskeletal was similar to the group in young women, being the most prevalent one. The metabolic-peripheral vascular group included conditions such as metabolic diseases, varicose veins, and cervical pain, and it was the second most frequent. We also found a neuropsychotic-endocrine cluster that included neurological and endocrine diseases, schizophrenia, personality and developmental disorders and had the highest mortality risk among young men. Finally, we found a visual deficit-behavior problems cluster that was the less prevalent group.

In men aged 45–64, five clusters were observed. The depressive-vascular-diabetic cluster, with conditions such as depression, varicose veins, diabetes, or ischemic heart disease, and the cardiometabolic cluster that included diseases like metabolic disorders, prostatic hypertrophy, ischemic heart disease, and arrhythmia, presented a similar mortality risk associated. We identified a neuro-substance use-cancer group that included diseases like substance use, neurological disorders, other respiratory disorders, high impact malignant neoplasms, or arrhythmia, and had the lowest prevalence and highest mortality risk associated. We also found a dermatological cluster that was associated with visual deficit disorders and dyslipidemia. In both men and women, the dermatological cluster included sensory deficits for the population aged 45–64. Finally, we found a dyslipidemic cluster that included dyslipidemia and low back pain and presented a similar mortality risk to the derma-visual deficit cluster.

In men aged 65 and older, we identified and described five clusters. The sensory-degenerative cluster included conditions such as degenerative joint disease, cataract, and dermatitis. The hypertensive cluster integrated by hypertension and diabetes, the cardiometabolic cluster with conditions such as dyslipidemia, hypertension, diabetes, and obesity, and the prostatic-cardiometabolic cluster, similar to the cardiometabolic one but including prostatic hypertrophy, all these three groups had similar mortality risk and prevalence. Finally, we also found a cardiovascular complex-degenerative group that had the highest mortality risk. The most frequent diseases in this cluster were arrhythmia, other respiratory disorders, congestive heart failure, and depression. It also included other chronic conditions like high impact malignant neoplasms, substance use, chronic ulcer of the skin, and malignant neoplasms of the lung.

We presented the complete output of the cluster analysis as supplementary material in which we included the complete disease analysis with their prevalence and prevalence ratios to compare the chronic conditions used to decide the composition of each cluster.

## Discussion

This study shows that multimorbidity affects three in every four patients with chronic obstructive airway diseases, revealing the existence of multimorbidity clusters in all age groups and both genders. Cardiovascular and metabolic diseases were amongst the most common conditions in many clusters. We identified associations of cardiometabolic diseases with mental health problems, dermatologic diseases with musculoskeletal disorders, and neurologic diseases with substance use disorders and neoplasms, amongst others. Epidemiological findings for each multimorbidity cluster, including prevalence and impact on mortality, could be a useful tool when planning healthcare interventions targeting patients with the most prevalent chronic obstructive airway diseases and multiple chronic conditions.

Our study revealed that multimorbidity is more common in patients with chronic obstructive airway diseases than in the general population and with a higher mean number of chronic diseases per patient^14^. This finding highlights the importance of implementing person-centered approaches in this population in clinical practice. The co-existence of conditions from different specialties in the same cluster constitutes a challenging situation for both clinicians and patients^14^. Interventions for the clinical management of populations with multimorbidity have focused primarily on other non-communicable chronic diseases than respiratory disorders^2^, and the small number of randomized clinical trials in patients with multiple chronic conditions results in uncertainty for the effectiveness of such interventions^15^. Disease clustering in the population with chronic obstructive airway diseases may provide valuable information for the development of educational activities on how to handle these patients.

Preventable cardiovascular risk factors such as hypertension, dyslipidemia, diabetes, and obesity were amongst the most common chronic conditions in various multimorbidity clusters, especially in the elderly. Metabolic syndrome is frequently associated with asthma, COPD, and OSA, but the nature of this relationship remains unclear^16^. In patients aged 45 and older, we found that metabolic diseases were associated with cardiovascular conditions in both genders; expected cardiometabolic clusters where COPD and OSA were especially prevalent. Systematic associations between COPD, diabetes, hypertension, and cardiovascular diseases such as cardiac arrhythmia and ischemic heart disease have been identified in adults over 45 years old^17^. These cardiovascular diseases share various common risk factors with COPD and OSA, like smoking and inflammation^5, 18^. Previous studies reported that a multimorbidity cluster of cardiovascular conditions, obesity, and diabetes in COPD patients with mild respiratory symptoms is associated with chronic low-grade systemic inflammation^19, 20^, and also poorer health outcomes^18^. Our findings showed that cardiovascular and cardiometabolic clusters have, in general, a higher likelihood of mortality, when compared with control groups. More studies are needed on this aspect in patients with chronic obstructive airway diseases and cardiometabolic comorbidities, considering the age of onset of the diseases and the treatments used. A recent study in OSA patients demonstrated the reduction of some inflammatory biomarkers after long-term treatment with CPAP and/or surgery of the upper airway in moderate-to-severe OSA patients^5^. Regarding COPD patients a large observational study reported that irrespective of history of exacerbations, new initiation of inhaled long-acting β2-agonists or antimuscarinic antagonists is associated with 1.5 times higher severe cardiovascular risk^21^.

Depression frequently co-exists in patients with respiratory disorders such as asthma and COPD^22, 23^; in our study, it was common comorbidity in clusters with cardiometabolic diseases. In women, depression was amongst the main conditions in two multimorbidity clusters, the dyslipidemic-depressive, and the depressive-degenerative clusters. In the last one, we found that depression was associated with hypertension, congestive heart failure, and degenerative diseases, with significantly high mortality risk. A recent study in COPD patients reported that cardiovascular disease is more prevalent in men, but its impact on mortality is higher in women, and that although depression is more common in women, its impact on mortality is similar in both genders^23^.

The MeDALL study found a strong association of asthma, allergic rhinitis, and eczema in children, suggesting the possible existence of an allergic cluster in the pediatric population^24^. Another study reported the existence of an allergic pattern in children, while in the population 15–29 years of age, growth and developmental disorders were also associated with asthma and dermatitis^14^. The same study found that in men 30–44 years old, this pattern included psoriasis. In patients 15–44 years old, we identified a derma-neurotic-musculoskeletal cluster with a high prevalence of allergic rhinitis and asthma among the chronic obstructive airway diseases and associated with dermatitis. This cluster also included psoriasis, kyphoscoliosis, and congenital anomalies, in both genders. Another relevant comorbidity in this cluster was anxiety and neuroses, and sensory-processing disorders (hearing loss in women and visual impairment in men). Further studies are needed to research the relationship between these associations, especially in the case of sensory-processing disorders, congenital anomalies and some musculoskeletal diseases in which there are very few studies reporting them^25, 26^.

Substance use disorder was the leading condition of the multimorbidity cluster with the highest likelihood of mortality in our study (ten times higher compared with the control group). It was identified in men 45–64 years old and included associations between neurologic disorders, cardiovascular diseases, and cancer, amongst other conditions, and had the highest prevalence of COPD. An unexpected cluster but with a logical sense. It is well established that tobacco is one of the main risk factors for COPD and lung cancer^27, 28^. Also, it has been reported that inhalation of heroin and cocaine by asthmatic patients increases asthma exacerbations and decreases pulmonary function^29^. Besides the direct deleterious effect of some substances, potential interactions with drugs should also be considered, especially in patients with multiple chronic conditions.

A recent study revealed significant drug–drug interactions in patients with respiratory diseases^30^; an example is the interaction of fentanyl with macrolides, which may increase the effect of the opioids. The importance of the present study is that it reveals unexpected systematic associations between chronic diseases, providing valuable information to generate hypotheses for potential physiological (including pharmacological) causal comorbidities. The presence of complex diseases in patients with multiple chronic conditions is challenging to assess, highlighting the importance of conducting further large-scale studies on the epidemiology of multimorbidity.

One of the principal strengths of our research is that it was conducted on a population-based cohort, including 98% of the reference population. We exhaustively studied the morbidity burden in patients with the most common treatable and preventable chronic obstructive airway diseases by including all chronic diseases recorded in electronic health records in both primary and specialized healthcare settings to enhance external validity. Besides, the diagnosis of chronic obstructive airway diseases was based on reports of hospital admissions and discharges with radiological confirmation of the event. Data in the EpiChron Cohort undergo continuous quality control checkups that ensure its accuracy and reliability for research purposes. One of the core limitations is the cross-sectional retrospective nature of the study, which does not allow us to know temporal characteristics, such as the age of onset of the diseases. We also lack lifestyle information, socioeconomic factors, information on functional status, and analytical variables, among others. Another limitation of this study is that we had no access to the information on spirometries to confirm the disease diagnosis, which could be especially relevant in COPD patients. Nevertheless, COPD diagnosis is Spain is based on the GOLD criteria^31^ and validated by the physicians in primary and hospital care. Furthermore, the use of spirometers in Aragon region has a penetration rate of 80–100% in primary care^32, 33^, with a higher proportion of trained healthcare professionals compared to other Spanish regions^32^. However, the number of spirometries performed in primary care is relatively low^33^, supporting the result that COPD patients in Spain have an estimated global under-diagnosis of 50–75%, which could implicate that some younger and older patients with COPD were not included in our study^34, 35^. This under-diagnosis could be the consequence of the interrelationship between primary care and hospital care, where preferably the spirometry is performed^33^. Nonetheless, an over-diagnosis of COPD is also possible. A cross-sectional study^36^ conducted in 2017 in the primary care setting in Spain reported an over-diagnose of COPD of 21%. We had to acknowledge this potential over-diagnosis of COPD in our population as a limitation of the study, which would contribute to an over-diagnosis of chronic obstructive airway diseases of approximately 4%.

## Conclusions

Multimorbidity affects almost three in every four patients with chronic obstructive airway diseases. The number and complexity of the identified multimorbidity clusters increase with age in both genders, revealing some unexpected systematic associations between diseases and a differential impact on mortality. The complex clinical profile of patients with the most prevalent chronic obstructive airway diseases highlights the need to implement holistic and person-centered clinical management approaches. Our findings may provide useful information when planning healthcare interventions targeting patients with chronic obstructive airway diseases and multiple chronic conditions. Further large-scale studies in different clinical settings and populations are encouraged to validate the results.

## Methods

### Study design and population

We conducted an observational retrospective study in the EpiChron Cohort. This cohort integrates, at the patient level, demographic and clinical information of the population assigned to the public health system in the Spanish region of Aragon (1.3 M individuals; approximately 98% of the total population of Aragon). The cohort profile, with a detailed description of data sources used, is published elsewhere^37^.

The study population was composed of patients 15 years of age and older with a diagnosis of at least one of the following chronic obstructive airway diseases registered in patients’ primary care electronic health records and/or hospital discharge reports in 2011: allergic rhinitis, asthma, emphysema, chronic bronchitis, COPD, and/or OSA. The identification of these entities was based on the diagnoses recorded in the clinical history of the patients and not on the pharmacological treatment received. This study was authorized by the Clinical Research Ethics Committee of Aragon (CEICA, PI16/0136). The CEICA did not require to obtain the patient's informed consent since all the information used was anonymized. We performed this research in accordance with the Declaration of Helsinki, as revised in 2013, and the Spanish Law on the protection of personal data (LOPD 15/1999 of December 14).

### Measurements

For each patient, we analyzed the following variables: age as of December 31, 2011, sex, place of residence (urban/rural), all diagnoses of chronic diseases, multimorbidity (defined as the simultaneous presence of two or more chronic diseases), and all-cause mortality during 2012–2014. We stratified age into three age intervals (i.e., 15–44, 45–64, and ≥ 65 years).

Diagnoses were coded initially according to the International Classification of Primary Care or to the International Classification of Diseases, in its Ninth Revision, Clinical Modification (ICD-9-CM), and subsequently grouped in Expanded Diagnostic Clusters (EDCs) according to the Johns Hopkins ACG System (version 11.0, Johns Hopkins University, Baltimore, MD, USA)^38^. We considered for the study the 114 chronic EDCs defined by Salisbury et al.^39^ as those diseases whose duration is ≥ 6 months, including the conditions of the past that require continued care and those at risk of recurrence. We also included allergic rhinitis in the analysis, as it is considered a chronic disease by the WHO^3^.

### Statistical analysis

We reported the clinical and demographic characteristics of the population with chronic obstructive airway diseases as means and/or frequencies, and differences according to sex were analyzed using the Chi-square test and the Wilcoxon test.

We used cluster analysis in each age and gender group to identify clusters of similar individuals according to their comorbidities. This technique allows assigning cases (i.e., patients) to groups so that those from the same cluster resemble each other more than those from other clusters in terms of comorbidity. Only patients with multimorbidity and diseases with prevalence higher than 1% were included in the cluster analysis. For each patient, we included in the analysis dichotomous variables representing the presence/absence of each of the comorbidities studied. The grouping unit was the individual^40^, with a total number of 97,759 patients included in the cluster analysis.

The selection of the cluster method followed an optimization process. First, we performed an agglomerative hierarchical method. However, this approach had computational limitations that required to perform the analysis in a sample of the population. For this reason, we finally decided to perform a k-means non-hierarchical analysis that allowed us to combine the statistical and clinical criteria for the selection of the number of clusters. We used the Jaccard coefficient as a measure of similarity because of the dichotomous nature of the diagnostic variables. This parameter establishes the distance between patients based on the number of observations (i.e., chronic conditions) they have in common and ignores the diagnoses that neither of them has. For the selection of the number of clusters, we aimed at maximizing model´s parsimony and we therefore looked for the lowest cluster number maximizing homogeneity within groups and heterogeneity between them and that had a consistent clinical interpretation. In this decision, we also considered the Calinski and Harabasz statistic to help to determine the optimal statistical number of clusters to be extracted in each group, which is indicated by the highest value of this statistic^41, 42^, however, this criterion was not always followed. The selection of the number of clusters and their clinical interpretation followed a process in which the clinical partners (J.C.-P., F.G.-R., I.I.-S., L.A.G.-M., J.D.-M., J.M.M., and A.P.-T.) were asked their opinion on the best option and discuss it in consecutive rounds if necessary. Thus, for each age and sex group, each clinical partner proposed the optimal number (and a second option if thought appropriated), and we valued all the opinions together until a consensus was reached.

We calculated the prevalence of each disease in each of the identified clusters. For each disease, we expressed prevalence differences among different clusters as prevalence ratios (PR). Prevalence and PR helped to define the disease composition of each cluster. A disease was initially included in a cluster if (1) the prevalence was higher than 25%, or (2) the PR was ≥ 2 in at least two-thirds of the comparisons with a prevalence higher than 2%, or (3) the PR was ≥ 1.5 in all comparisons and the prevalence was higher than 2%. In the case of doubts, the decision was made based on the clinical interpretation of the cluster according to common pathophysiological mechanisms, clinical relevance, and prevalence. The denomination of the clusters was agreed according to the most relevant diseases within each pattern, considering their prevalence, prevalence ratio, and clinical relevance, and the denominations already used in the literature.

For each cluster, we performed an age-adjusted logistic regression analysis to determine if the mortality (%) was different between clusters taking as reference the group with chronic obstructive airway diseases and no multimorbidity. We conducted all the analyses in STATA software (Version 12.0, StataCorp LLC, College Station, TX, US), and statistical significance was established at *p* < 0.05.

## Supplementary information

Supplementary information.
